# Optical assembly of bio-hybrid micro-robots

**DOI:** 10.1007/s10544-015-9933-1

**Published:** 2015-02-15

**Authors:** Álvaro Barroso, Shirin Landwerth, Mike Woerdemann, Christina Alpmann, Tim Buscher, Maike Becker, Armido Studer, Cornelia Denz

**Affiliations:** 1 Institute of Applied Physics, Westfälische Wilhems Universität, Correnstrasse 2-4, 48149 Muenster, Germany; 2 Organic Chemistry Institute, Westfälische Wilhems Universität, Correnstrasse 40, 48149 Muenster, Germany

**Keywords:** Bio-hybrid systems, Micro-robots, Holographic optical tweezers, Non-spherical shape, Zeolite L nanocontainers, Micromanipulation

## Abstract

The combination of micro synthetic structures with bacterial flagella motors represents an actual trend for the construction of self-propelled micro-robots. The development of methods for fabrication of these bacteria-based robots is a first crucial step towards the realization of functional miniature and autonomous moving robots. We present a novel scheme based on optical trapping to fabricate living micro-robots. By using holographic optical tweezers that allow three-dimensional manipulation in real time, we are able to arrange the building blocks that constitute the micro-robot in a defined way. We demonstrate exemplarily that our method enables the controlled assembly of living micro-robots consisting of a rod-shaped prokaryotic bacterium and a single elongated zeolite L crystal, which are used as model of the biological and abiotic components, respectively. We present different proof-of-principle approaches for the site-selective attachment of the bacteria on the particle surface. The propulsion of the optically assembled micro-robot demonstrates the potential of the proposed method as a powerful strategy for the fabrication of bio-hybrid micro-robots.

## Introduction

Linking molecular motors of biological cells with inorganic micro- and nanoscale components represents nowadays one of the most versatile ways to produce miniature mobile robots designed to operate in a liquid medium (van den Heuvel and Dekker 2007). In comparison to the smallest man-engineered machines (Dreyfus et al. 2005; Donald et al. 2006; Zhang et al. 2009; Nelson et al. 2010; Fischer and Ghosh 2011; Zhou et al. 2014; Mark et al. 2013), these so-called *bio-hybrid micro-robots* or *living micro-machines* have key advantages which promote them as exciting tools for biomedical and nanotechnological applications. On the one hand, biomolecular motors directly convert chemical energy from the surrounding medium to mechanical energy with high efficiency. This on-board actuation enables to reduce the size of the machine, and thus to overcome the challenge for miniaturization of the energy power source. In addition, they are not expensive, do not require bulky instrumentation for production and can be modified through genetic engineering (Diez et al. 2005). On the other hand, the employment of micro- and nanocontainers as carriers of drug molecules or as agents for sensing and imaging applications permits to envision these bio-hybrid living machines as futuristic tools for on-board diagnosis and therapy (Sitti 2009).

The rotary nanomotors of flagellated bacteria are the most exploited biological motors employed for actuation and powering devices (Martel 2012) because of their high efficient mechanism for motion in the low Reynolds number regime, where viscous forces dominate over inertial forces. For propulsion, motile bacteria such as *Escherichia coli* and *Bacillus subtilis* feature one or more locomotive appendages called flagella which consist of a stiff helical protein filament that is rotated by a motor embedded in the cell wall and a hook which connects the motor to the filament (Berg 2000). When the filaments rotate in synchronization, they form a coherently rotating bundle that propels the cell forward. This “run” mode alternates with the “tumble” mode when one or more flagella motors reverse their rotation, leading to a change in the direction of the bacterium’s motion (Darnton et al. 2007). The resulting three-dimensional random walk has been used to propel particles, reaching from conventional microspheres to more complex abiotic objects as non-spherical micro-particles and to rotate microscopic gears (Darnton et al. 2004; Sokolov et al. 2010; Sakar et al. 2011). More importantly, when used as micro-actuators, bacteria can be steered *via* various mechanisms or taxes influencing their tumbling frequency in such a way that they can transport the payload particle of the bio-hybrid assembly to specific locations. For instance, magnetotactic bacteria has been used to actuate micro-objects by means of off-board magnetic fields (Martel et al. 2006), and chemotactic bacteria have been employed to propel particles in direction of favorable nutrients (Kim et al. 2012). With the idea of performing designed biomedical tasks, attention has shifted recently to more functional objects capable of carrying and delivering therapeutic and diagnostic agents. In this respect, porous materials are highly interesting because they can accommodate guest molecules. Zeolites L are framework aluminosilicates containing one-dimensional nanochannels able to accommodate a variety of molecules and constitute a nontoxic nanotransporter that can be functionalized on its surface and on the channel entrances. Zeolite L crystals serve thus as a very suitable building block to form bio-hybrid micro-robots: whereas the interior nanochannels of zeolite-L crystals can be loaded with drug molecules or luminescent dyes for imaging and labeling biological cells, the outer surface can be chemically modified to allow assembly and targeting of the bacteria (Popović et al. 2007; Strassert et al. 2009). Furthermore, zeolite L crystals can be synthesized with various defined morphologies and sizes raging from 30 nm up to 10 of micrometer (Lee et al. 2005, Calzaferri 2012).

Established methods for the formation of bio-hybrid micro-robots rely on the uncontrolled coupling of the abiotic particle and the biological specimen in solutions. A lot of progress has been made to tackle this problem by the site-selectively surface functionalization of the abiotic object which leads the biological specimen to attach only where the surface has been modified (Popović et al. 2007; Behkam and Sitti 2008; Cho et al. 2012). Nevertheless, the demonstration in the microrobotic field of robust methods which allow controlling the number of bacteria and precisely attaching them on the particle surface is still lacking. Optical tweezers is a well established technique which enables to move transparent microscopic particles which are confined near the focus spot of a tightly focused laser beam by means of light gradient forces (Ashkin et al. 1986). Due to their versatility to manipulate in a contactless and aseptic way particles with a higher refractive index than the surrounding environment, as it is the case for cells and bacteria cultures, optical tweezers have become a standard technology for manipulating and controlling biological objects (Svoboda and Block 1994). For example, simple optical tweezers have been used to position bacteria at surfaces in a predetermined area (Haruff et al. 2003; Woerdemann et al. 2014) and to investigate the adhesion of bacteria to inorganic surfaces, as *e.g.*, glass (Jones and Velegol 2006) and functionalized coated microspheres (Simpson et al. 2002). However, aiming to assist the formation of a bio-hybrid system that consists of at least two building blocks requires the use of more than one single optical trap. This challenge can be finely addressed with *holographic optical tweezers* (HOT). This approach extends the concept of optical tweezers to the dynamic manipulation over a multitude of particles by tailoring the light trapping beam into several single tweezers or even more complex optical traps as *e.g.*, non-diffracting and self-similar light fields (Woerdemann et al. 2012). In this proof-of-principle study, we demonstrate the versatility of holographic optical trapping for the controlled fabrication of a single bacterium and a single abiotic particle. As an important demonstration example, we employ zeolite L nanocontainers as carriers and motile *Bacillus subtilis as* the propelling living organism. We discuss various schemes to perform the optical assembly of the bacterial micro-robot and finally we demonstrate the validity of our method by showing the propulsion of the abiotic particle by the optically attached bacterium. Our concept presents for the first time the simultaneous observation and fabrication of living micro-robots just by optical means, and thus opens new perspectives for the fabrication of these systems in a contactless and minimal invasive way.

## Experimental section

### Holographic optical tweezers setup

**Fig. 1:**
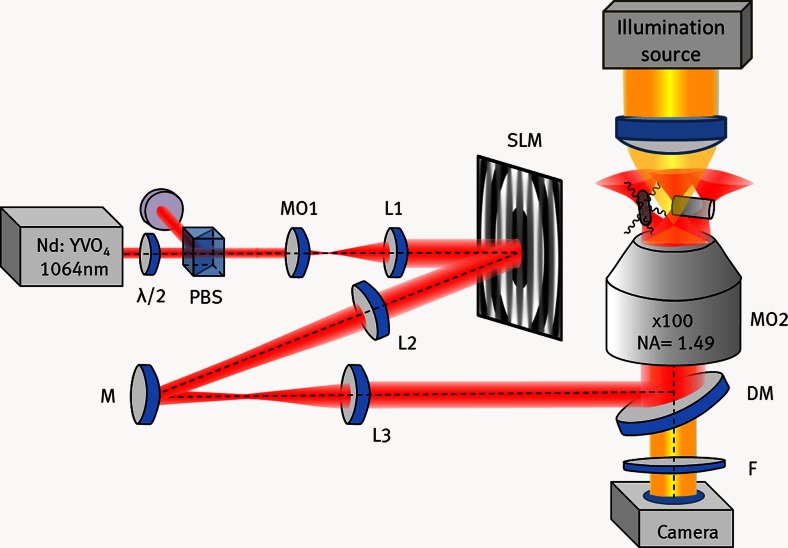
Sketch of the HOT system. λ/2: half wave plate; PBS: polarizing beam splitter cube; MO1,2: microscope objective; L1-L3: lenses; SLM: spatial light modulator; M: mirror; DM: dichroic mirror; F: filter; NA: numerical aperture

For observation and optical manipulation, we used an in-house developed HOT system that was implemented on the basis of a commercially available inverted fluorescence microscope (Eclipse Ti, Nikon) with a high numerical aperture microscope objective (Nikon Apo TIRF, 100×/1.49 Oil-immersion). Fig. 1 illustrates a schematic of the HOT experimental setup, which is described in detail in (Woerdemann et al. 2012). The HOT system used as trapping light source a Nd:YVO_4_ laser (Smart Laser Systems, Berlin, Germany) operating at a wavelength of λ =1064 nm and of maximum output power 2.5 W. A half-wave plate (λ/2) and a polarizing beam splitter (PBS) were placed at the beginning of the optical path in order to adjust the laser trapping power which was set to 200 mW measured at the back aperture of the inverted microscope. A beam expander, formed by microscope objective MO1 and lens L1, was used to illuminate the whole surface of a phase-only spatial light modulator (SLM) (1920 × 1080 pixels, “Pluto” LCOS display from Holoeye Photonics, Berlin, Germany). The SLM was used as a diffractive optical element to tailor the light field in several Gaussian shaped optical tweezers and to steer them three-dimensionally by displaying the phase pattern corresponding to the desired optical trapping configuration in the sample plane of the microscope (Liesener et al. 2000). For calculation of the phase pattern, we adapted a free software that was originally developed at the University of Glasgow, Scotland (Bowman et al. 2010). The SLM was imaged *via* a telescope, formed by lenses L2 and L3, onto the back aperture of the high numerical microscope objective MO2. A dichroic mirror (DM) was used to reflect the laser beam into the microscope objective MO2 and a filter (stop band at λ = 1064 nm) was used to separate the laser beam path from the observation path. The calculation of the holograms displayed in the SLM was performed with video repetition rate by using adequate computer hardware and thus providing dynamic on-line manipulation of optically trapped objects. Images of the assembly process and the subsequent motion of the bio-hybrid machine were acquired by a Photonfocus camera (MV2-D1280-640-CL). For determination of position of the living micro-robots, manual tracking at each frame was performed.

### Models of abiotic and biological system

Cylindrical-shaped zeolite L crystals of 2 μm of length, base area of 1 μm of diameter, and empty channels were used as model systems of the abiotic particle. This particular aspect ratio was chosen because zeolite L crystals featuring a strong asymmetry are easier to be fully controlled in three dimensions with HOT (Woerdemann et al. 2010). Furthermore, when actuated by swimming bacteria, elongated non-spherical particles have been reported to have a higher degree of directionality due to the high resistance to rotation around the short principal axes of the particle (Sahari et al. 2012). As a model of swimming prokaryotic cells, we chose a wild type strain of rod-shaped *B. subtilis* (BD 360) which have 0.8 μm in diameter and 2 μm length approximately. *B. subtilis* is a Gram-positive bacterium featuring 2–9 flagella filaments distributed all over the body which propel the cell through the surrounding medium. Due to their elongated body geometry and their swimming velocities, *B. subtilis* can be also elegantly controlled with few miliwatts per optical trap of the HOT (Hörner et al. 2010).

In the size range of the employed building blocks that were used to form the bio-hybrid machine, assemblies can be produced on the basis of colloidal interactions (Benito et al. 2008). As it is the case for Gram-positive bacteria, *B. subtilis* presents a negatively charged cell membrane (Hori and Matsumoto 2010). On the other hand, zeolite L crystals have a framework where each aluminum atom contributes with a negative charge at their surface which is compensated by the presence of cations within their structure. Under these physiological conditions, no electrostatic attraction exists between the bacteria and the zeolites surfaces and therefore bacterium-zeolite L adhesion may not occur. Advantageously, the surface of zeolite L crystals can be site-specifically chemically modified in order to positively charge the surface, while the inner structure of the crystal remains unaltered. Based on the results of former experiments reporting on the non-covalent binding of zeolite L crystals to bacteria (Popović et al. 2007; Strassert et al. 2009), we coated the whole surface of our zeolites with amino groups to favor their adhesion to the bacterial surface. The zeta potential obtained for *B. subtilis* and for zeolites L crystals without and with amino groups amounted to −37.0 mV, −77.3 mV and 17.1 mV, respectively, and thus support that cell adhesion might occur only for the amino-functionalized zeolites.

### Environmental conditions for assembling the bio-hybrid living machine

When immersed in a medium of high ionic strength, zeolite L crystals adhere effectively to each other or on cover glass surfaces (Veiga-Gutiérrez et al. 2012). We observed that when prepared in the salt-rich medium of bacteria, zeolite L crystals adhered to the thin microscope slide cover glass after a few minutes in suspension when the crystals were located near the surface. Thus, to establish an environment with an optimal supply of crystals to perform the assembly, zeolite L crystals and bacteria were prepared in two connected reservoirs allowing the transport of particles from one to another. In one of the reservoirs, zeolite L crystals were suspended in deionized water with 1 % of a non-sticking biocompatible surfactant (Tween 20, Carl Roth, Germany). In the second, we provided *B. subtilis* in a chemotaxis buffer which enabled appropriate smooth swimming of the bacteria. Major part of the bacteria showed the typical running and tumbling behavior with running velocities between 1 and 30 μm/s. The optical assembly and subsequent transport of the particle were performed in the reservoir of the bacteria. However, due to actuation by the bacteria, the dwell times were not long enough for the zeolite to get adhered on the surface of the cover glass.

**Fig. 2:**
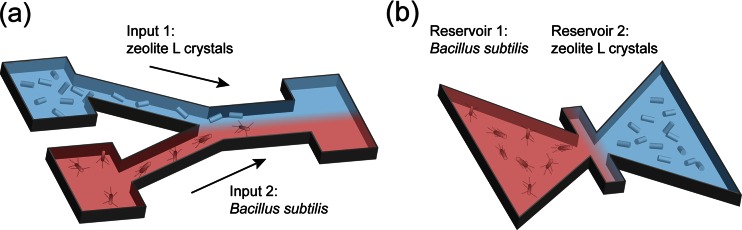
Experimental schemes used for the assembly of the living micro-robot. **a** Y-shaped PDMS microchannel with an input supplying zeolite L crystals and a second input providing the bacteria in a chemotaxis buffer. **b** Petri dishes with two defined reservoirs containing each the bacteria and the zeolite L crystals

Two different schemes having the features described above were used for the experiments. A schematic representation of the two devices is depicted in Fig. 2. The first approach consisted of a home-made Y-shaped PDMS microchannel system consisting of two independent microfluidic pumps supplying an input with the surfactant-rich solution of zeolites and another with bacteria in the chemotaxis buffer at an approximately flow rate of 5 nl/s. For the second approach, we used commercially available Petri dishes which have two defined reservoirs and a center line to operate the assembly (μ-Slide Chemotaxis 3D, ibidi GmbH, Germany). For our goal, both approaches were tested to be equally valid, although the constant supply of particles in the first approach may be more suitable if automation of the optical assembly is desired.

## Optical construction of the living micro-machine

A main advantage of using HOT to fabricate assemblies of microparticles is its ability to manipulate individually the building blocks of the system while the assembling process can be simultaneously observed and recorded. This allows controlling the relative position between the blocks forming the assembly, which in perspective enables the manufacturer to optimize the design of the micro-robot. Here we present two proof-of-principles schemes for the construction of living micro-robots consisting of a single elongated zeolite L crystal and a single bacterium. In a first model experiment a method for fine positioning the bacterium on the abiotic particle is presented. In a second scheme we present a coarse method which allows for an effective assembly of fast swimming bacteria, but, on the other hand, a lower defined control on the relative position between the abiotic and biotic particles.

### Fine site-specific attachment

**Fig. 3:**
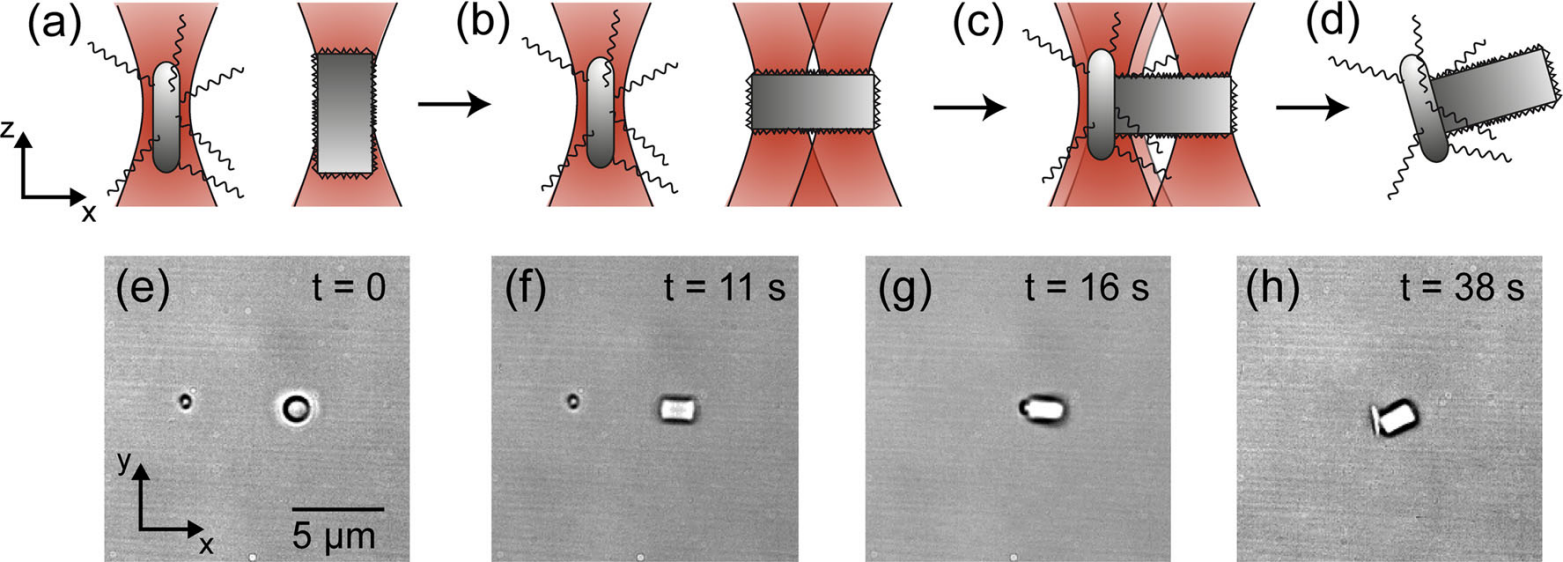
(**a**-**d**) Schematic and (**e**-**h**) corresponding demonstrative experimental microscopy images of the steps and optical trap configurations for the fine attachment of a single bacterium on a specific area of the surface of a zeolite L crystal

Figure 3 shows schematic and corresponding experimental images of the steps towards the fine attachment with HOT of a single bacterium on a specific site of the cylindrical zeolite L. This exemplary case was performed with the Y-shaped microfluidic channel which is illustrated in Fig. 2a. The process to construct a bio-hybrid micro-robot consisted of the following steps. First, a single zeolite L crystal that was located in the reservoir containing non-sticking medium was trapped with one single laser beam and approached to the other reservoir by moving the microscope translation stage. The refractive index difference between the aqueous medium (n_medium_ ≈ 1.33) and the zeolite L crystal (n_zeolite_ ≈ 1.5) resulted in a high trapping efficiency and ensured that the zeolite L crystal remained in the optical trap during the translation to the bacteria’s reservoir. In a second step, the trapping laser beam was split in two optical traps by displaying the appropriate hologram on the SLM. The second optical trap was used to trap a bacterium swimming within the microscope field of view (Fig. 3a). The optical trapping of bacteria was also very stable, although very motile bacteria may escape from the optical trap if the optical forces do not counterbalance the bacterial locomotive forces. Owing to their elongated shape, both particles inherently align along the z-axis, *i.e.*, along the direction of propagation of the optical trapping beam (Simpson and Hanna 2011). Consequently, both particles were observed as circular spots with the microscope (Fig. 3e). However, a beneficial feature of HOT is that they enable to rotate precisely elongated particles with respect the optical axis by creating an asymmetric potential with multiple traps. In this way, the whole elongated particle can be observed laterally. In our approach, the full control of the particle’s orientation allows to position the bacterium specifically in an arbitrary surface of the zeolite L crystal. Thus, in order to demonstrate the versatility of our method in the next step a third optical trap was created to position one of base area of the crystal facing the bacterium. For this purpose, the third trap was initially superimposed to the existing optical trap holding the zeolite and used as handle to rotate the elongated zeolite by smoothly steering 2 μm the optical trap in the x-direction (Fig. 3b). As a result of the displacement, a torque is applied in one extreme of the zeolites which enables to rotate it 90 degrees respect to the optical axis (Fig. 3f). The new configuration was very stable, remaining the particle in this position as long as required (Woerdemann et al. 2010). At this step, control over the particles within sub micrometer precision was required to position the bacterium in a specific base area of the zeolite. Therefore, the approaching of the bacterium to the zeolite was carried out by moving the transversal position of the optical traps by modifying the phase pattern that was displayed in the SLM. When the bacterium and the zeolite L crystal were observed to be in close contact, they were trapped few more seconds with the optical tweezers to ensure that, despite the bacterium motion within the optical trap, both surfaces indeed electrostatically interacted (Fig. 3c, g). Finally, when the trapping laser was turned off, both parts remained together and hence demonstrate the optical assembly of the bio-hybrid micro-robot (Fig. 3d, h). After performing the assembly, we observed that neither by using two optical traps to separate the bacterium from the zeolite nor by dragging the zeolite with the optical tweezers became the bio-hybrid machine disassociated. The reproducibility of the method depended highly in the motility behavior of the bacteria. In this way, high motile bacteria were difficult to trap with the optical tweezers and thus the optimal bacteria to be used for the bio-hybrid assembly were those which showed a relative slow movement (v < 10 μm/s). Also, it has to be noted that in some cases the functionalization of the zeolite L crystals might be not equally distributed all over the surface of the crystal, resulting in a decrease of the effectiveness of the assembly process.

### Coarse site-specific attachment

**Fig. 4:**
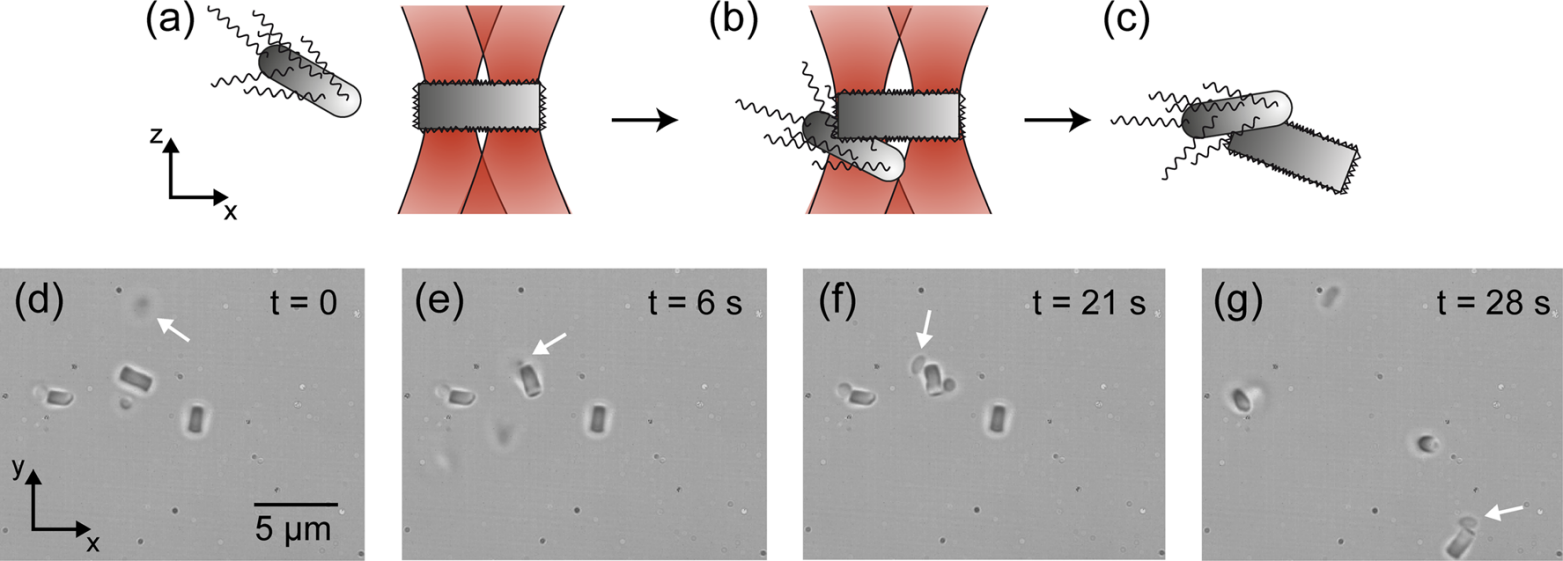
Schematic (**a**-**c**) and corresponding experimental microscopy images (**d**-**g**) of the steps and optical trap configurations for the coarse attachment of a single bacterium (*marked with a white arrow*) on a specific area of the surface of a zeolite L crystal

When bacteria motility might hamper the performance of the optical assembly by the fine method, it can be alternatively exploited for the fabrication process of the bio-hybrid micro-robot. In such cases, a rougher and faster approach could be also applied to assemble a bacterium on the surface of a zeolite L. Figure 4a-c show schematics of the assembling process of a bio-hybrid machine by the coarse-attachment method. The method consists of horizontally aligning several zeolite L crystals with two optical traps as it has been described in section 3.1. When a swimming bacterium approaches an optically trapped zeolite L, it is trapped by one of the optical traps holding the zeolite and subsequently attached closely to one of the base zeolite’s surfaces.

Figure 4d-g show images of the assembly process of a bacterium-zeolite L living machine by this approach. In this particular example, the second experimental scheme (Fig. 2b) was used and three zeolite L crystals were optically trapped and transported from the zeolite’s reservoir to the bacteria’s reservoir. In t = 0 these zeolites were optically arranged horizontally each with a pair of optical traps situated in the base area of the cylinders (Fig. 4d). The use of more than one zeolite increased the probability to catch a self-propelling bacterium. In addition, the horizontal alignment of the zeolites favor that swimming bacteria were likely to get attached near the base area of the zeolite (Fig. 4e-f). Once a bacterium was trapped, the bacterium was retained for few seconds in the optical trap in order to let the bacterium-zeolite L surfaces interact. As the trapping laser was turned off, the subsequent transport of the zeolite with the bacterium attached to it demonstrates the assembly of the bio-hybrid machine (Fig. 4g). Skipping the step for positioning finely the bacterium on the surface of the zeolite enables to reduce the time for the assembly to half a minute. However, in both cases the required time to assemble the micro-robot is very below the reported time (20 min) at which optical tweezers might cause physiological damage to bacteria (Rasmussen et al. 2008).

### Transporting the load

**Fig. 5:**
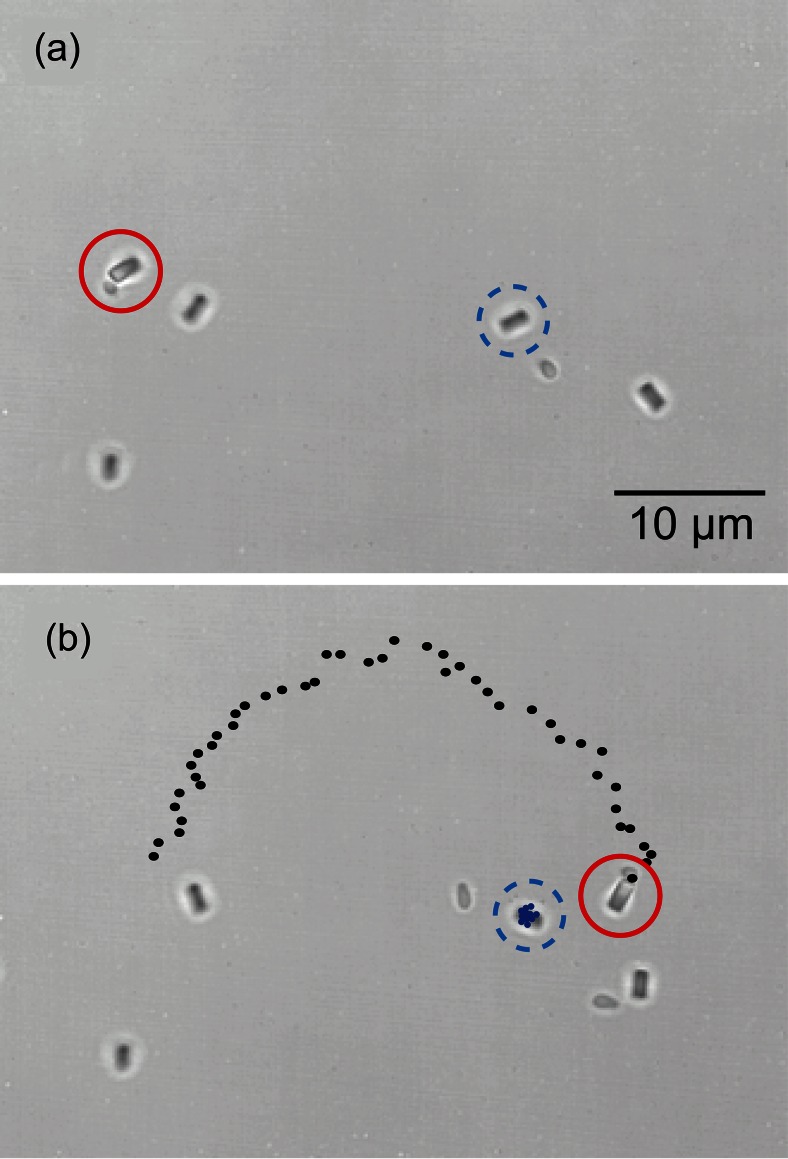
Microscopy images illustrating a swimming micro-robot made of a single bacterium and a zeolite L crystal (*red circle*) and suspended zeolite L crystal (*dashed blue circle*). Time lapse between images is 6 s. In (b) the corresponding representative trajectories of the micro-robot and the suspended zeolite crystal are marked with black points and blue points, respectively

Having developed the schemes to construct a bio-hybrid system formed by a single living bacterium and an elongated zeolite L crystal, whether by the fine or the course approach, it is crucial to demonstrate the propulsion of the particle by the bacterium. Figure 5 shows white light transmission microscopy images of a bacterium-zeolite L machine which is surrounded by some swimming bacteria and zeolites in suspension in the chemotaxis buffer. Figure 5b illustrates also the particle trajectories of the bio-hybrid living machine and one of the suspended zeolites whose motion was driven by Brownian diffusion. In an interval of time of 6.6 s, the distance covered by the living machine was about 45 μm. Then, the averaged velocity was calculated to be 7 μm/s approximately. A comparison between the trajectories of the living machine (black point trajectory) and the suspended zeolite L crystal with no bacterium attached on the surface (blue point trajectory) serves as a control experiment to demonstrate the propulsion of the zeolite by the bacterium.

As it has been described in the introduction section, wild-type *B.subtilis* bacteria typically show a random succession of directional motion (“run”) and erratic, random rotation (“tumble”). Interestingly, we observed that the trajectories of bacterium-zeolite L micro-robots do not feature the characteristic straight trajectories of these self-propelling bacteria during its running mode (velocity of this exemplary bacterium during its run phase was 4 μm/s approx.), but show a curved trajectory which might be explained by the asymmetric configuration of the bacterium-zeolite machine. Moreover, during the propulsion, the zeolite L crystals of the bio-hybrid assembly did not attach to the cover glass surface, although being contained in a salt-rich chemotaxis buffer. Additionally, our experimental scheme is fully compatible with the use of chemical gradients which, as demonstrated in (Kim et al. 2012), can direct the motion of the living machine towards the direction of a chemoattractant source.

## Conclusion

The fabrication of micro-robots that are propelled by bacterial flagella represent a promising strategy for future biomedical applications, with a disruptive potential especially concerning minimally invasive diagnosis and localized treatment of diseases. In this proof-of-principle study, we have presented a novel approach based on holographic optical trapping for the assembly of bio-hybrid micro-robots. In comparison with self-assembling schemes, our approach has the particular ability to control just by optical means the construction of micro-robots consisting of a single self-propelling bacteria and one abiotic particle. With HOT, tailored optical trap configurations can be created for the parallel manipulation in three dimensions of elongated microcrystals and rod-shaped bacteria. Owing to the sub micrometer precision of HOT, the single bacterium can be attached with a high degree of accuracy on the surface of the payload particle. We also show that typical duration of the optically assisted assembly requires much less time than that reported to induce photo damage on the bacteria specimen with the optical trapping laser. This is demonstrated by the subsequent propulsion of the abiotic particle by bacterial cells with a velocity similar to other reports (Darnton et al. 2004; Behkam and Sitti 2007).

Based on the advance of holographic optical manipulation techniques, our approach paves the way to a custom design of bio-hybrid micromachines. In this respect, our concept offers unique opportunities to create more complex machines, as *e.g.*, micro-robots with a specific number of optically arranged bacteria and abiotic particles. It will also facilitate the study of the movement of the bio-hybrid machines in dependence of the position of the payload particle and the propelling bacterium. In principle, the method can be applied to any type of functional particles and bacteria which can be driven by other mechanisms, as magnetotaxis or phototaxis. Moreover, the simultaneous assembly and the implementation of our approach in microfluidic devices could permit the automatized construction of armies of micro-robots. Finally, the unidirectional channels of zeolite L crystals could be used for selective transport and release of bioactive molecules, as *e.g*., antibiotics and drugs. In conclusion, we believe that the contactless and spatiotemporal controlled optical assembly of bio-hybrid micro-robots opens up future possibilities for the fabrication of such systems and will expand the field of biomedical microrobotics.
